# Bottom-up transdiagnostic personality subtypes are associated with state psychopathology: A latent profile analysis

**DOI:** 10.3389/fpsyg.2023.1043394

**Published:** 2023-02-21

**Authors:** Helo Liis Soodla, Kirsti Akkermann

**Affiliations:** ^1^Institute of Psychology, University of Tartu, Tartu, Estonia; ^2^Centre for Cognitive and Behavioural Therapy, Tartu, Estonia

**Keywords:** personality subtypes, latent profile analysis, impulsivity, perfectionism, transdiagnostic, dimensional psychopathology, dimensionality

## Abstract

**Introduction:**

Personality-based profiling helps elucidate associations between psychopathology symptoms and address shortcomings of current nosologies. The objective of this study was to bracket the assumption of *a priori* diagnostic class borders and apply the profiling approach to a transdiagnostic sample. Profiles resembling high-functioning, undercontrolled, and overcontrolled phenotypes were expected to emerge.

**Methods:**

We used latent profile analysis on data from a sample of women with mental disorders (*n* = 313) and healthy controls (*n* = 114). 3–5 profile solutions were compared based on impulsivity, perfectionism, anxiety, stress susceptibility, mistrust, detachment, irritability, and embitterment. The best-fitting solution was then related to measures of depression, state anxiety, disordered eating, and emotion regulation difficulties to establish clinical significance.

**Results:**

A 5-profile solution proved best-fitting. Extracted profiles included a high-functioning, a well-adapted, an impulsive and interpersonally dysregulated, an anxious and perfectionistic, and an emotionally and behaviorally dysregulated class. Significant differences were found in all outcome state measures, with the emotionally and behaviorally dysregulated class exhibiting the most severe psychopathology.

**Discussion:**

These results serve as preliminary evidence of the predictive nature and clinical utility of personality-based profiles. Selected personality traits should be considered in case formulation and treatment planning. Further research is warranted to replicate the profiles and assess classification stability and profiles’ association with treatment outcome longitudinally.

## 1. Introduction

Comparative investigations of categorical and dimensional psychopathology classification systems highlight the need to transcend a binary disordered-healthy division (Dalgleish et al., 2020). Firstly, wide-spread comorbidity, apparent in diagnostic fluidity and co-occurrence of disorders, complicates differentiation between primary and secondary diagnoses (Stice et al., 2013; Steinert et al., 2014; Lai et al., 2015; Bahji et al., 2019). Besides diagnostic overlap, within-disorder heterogeneity hinders conceptualization (Fried and Nesse, 2015). Conjointly, there is evidence of a mismatch between categorical assignment of diagnoses and dimensionality of symptoms (Beesdo et al., 2010; Sysko et al., 2010). This is exemplified by occurrence of sub-threshold impairments and “not otherwise specified” disorders (Regier et al., 2013). Since categorization without accounting for shared underlying variability can be arbitrary, current classification practices can result in hinderances in treatment planning (Chmielewski et al., 2015; Hopwood et al., 2020).

One way of grounding classification is to consider personality traits as basis for drawing distinctions — these patterns of cognition, emotion, and behavior dispose individuals to pathology, impact treatment adherence and prognosis (Andersen and Bienvenu, 2011). Searching for traits contributing to the etiopathogenesis of mental disorders and drawing from the rich discourse on theoretical and empirical personality typologies, Krueger and Eaton (2010) highlight antagonism, disinhibition, negative affectivity, introversion, and peculiarity, reflecting impairments in general affectivity, impulse control, and interpersonal functioning. Empirical evidence underscores these traits. Negative affectivity has been shown to characterize depression, anxiety and substance use disorders (SUDs; Kotov et al., 2010; Paulus et al., 2015). Impulsivity has been related to suicidality, anger, hostility, mood, and anxiety disorders and eating disorders (EDs; Johnson et al., 2013; Santens et al., 2020), while overcontrol and perfectionism, a common proxy, have been connected to EDs, social anxiety disorder, generalized anxiety disorder (GAD), post-traumatic stress disorder (PTSD) and panic disorder (Egan et al., 2011). Interpersonal dysregulation has been shown to be a vulnerability factor in SUDs, anxiety, and mood disorders (Koob and Volkow, 2010; Judd et al., 2013; McEvoy et al., 2013). Additionally, these characteristics mirror the maladaptive traits that, together with deficits in general functioning, constitute personality disorders under the DSM-5 Alternative Model (Krueger and Hobbs, 2020).

Recognition of such traits’ transdiagnostic influence has given rise to personality-based profiling. Bohane et al.’s (2017) systematic review revealed most clinical profiling studies to have focused on individuals with EDs. Studies have converged on a 3-class solution that encapsulates ideas first highlighted in Block and Block’s (1980) theory of ego resilience and ego control: undercontrolled, overcontrolled and resilient profiles arise (Wildes and Marcus, 2013). The initial undercontrol-overcontrol designation in Robins et al.’s (1996) seminal study describes the undercontrolled class as stubborn, impulsive, externalizing and restless, while the overcontrollers are inhibited, introverted, restrictive and internalizing (for a review of the framework’s applications, see Bohane et al., 2017). The semantic scope of these personality constructs has expanded, yet the robust tripartite model holds in clinical samples: in ED studies, the undercontrolled class has been characterized by emotional lability and behavioral disinhibition, the overcontrolled by avoidance, emotional restriction, rigid perfectionism, and anxiousness; the resilient class has been shown to function well and demonstrate moderate levels of perfectionism and conscientiousness (Westen and Harnden-Fischer, 2001). Among clinical populations, comparable results have emerged in research on people with PTSD (Thomas et al., 2014). These three personality prototypes have also been cross-culturally postulated in healthy control samples (e.g., Asendorpf et al., 2001; Rammstedt et al., 2004). As demonstrated by Christian et al. (2021), impulsivity- and perfectionism-based profiles are also differentially related to clinically significant outcome variables among non-clinical samples. High perfectionism was associated with compulsivity, worry, social anxiety, depression, restricting and bingeing; high impulsivity with pronounced alcohol use; while a combination of high perfectionism and impulsivity was related to both internalizing and externalizing psychopathology (Christian et al., 2021).

To accommodate more heterogenous samples, this model has been repeatedly refined. For example, in clinical populations either combined classes of perfectionism and impulsivity or several classes of over- or undercontrolled participants have surfaced (Boone et al., 2014; Soidla and Akkermann, 2020). Similarly, on large population-based samples, there is evidence for five-to seven-class solutions (Herzberg and Roth, 2006; Eaton et al., 2011; Sava and Popa, 2011).

As expected, the prototypical overcontrol-undercontrol profiles are less distinctly differentiated in these more diverse clinical samples. While population-based samples point toward the possibility of widely applying the tripartite classification (see, e.g., Yin et al., 2021), evidence for such distinctions within affective disorders—anxiety and mood disorders—is mixed (Spinhoven et al., 2012; Ulbricht et al., 2018). Note-worthy transdiagnostic classification research includes Brown and Barlow’s (2009) studies on depressive and manic mood, autonomic activation, intrusive cognitions, social well-being, avoidance, and trauma. These traits were found to make up six personality profiles: two classes with limited impairment, a panic-somatic (elevated autonomic arousal and somatic anxiety), a social-depressed (depressive mood and anxiety in social situations), an obsessed-worried (intrusive thoughts) and a severe-comorbid class, with heightened impairment on all measures (Rosellini and Brown, 2014). These results point toward the utility of including additional personality traits, reflecting general affectivity and interpersonal functioning, to increase explanatory power and clinical utility.

Compensating for the underexplored niche of mixed clinical samples, the Hierarchical Taxonomy of Psychopathology (HiTOP) synthesized previous studies and postulated an internalizing spectrum comprising mood and anxiety disorders, and EDs (Kotov et al., 2017; Dalgleish et al., 2020). Individuals with syndromes on the internalizing spectrum share dysfunctional personality traits like anxiousness, hostility, and emotional lability, which can be interpreted as facets of negative affectivity and irritability (Kotov et al., 2017). However, HiTOP and previous investigations of personality profiles and transdiagnostic traits diverge in two aspects. Firstly, in the context of generalizing from ED studies to the general psychiatric population, HiTOP fails to sufficiently recognize the variance of both impulsivity and perfectionism within the internalizing spectrum—urgency and low behavioral control are listed as traits of the externalizing spectra (Kotov et al., 2017). Furthermore, SUDs are identified as a distinct subfactor on the externalizing spectrum, offering little explanation for their co-occurrence with mood and anxiety disorders and EDs (Blanco et al., 2015).

Review of previous research reveals two methodological imbalances. Firstly, studies often either address specific diagnostic classes or the entire psychopathological nosology. In the former case, joint transdiagnostic basis is ignored, failing to allow for cross-diagnosis clusters and challenge current classification. In the latter, associations between extracted clusters can remain unclear. Secondly, exploratory and confirmatory approaches do not build on each other: hypotheses-free studies hinder progress by refraining from comparing nosologies and re-assessing existing models on new samples; confirmatory studies can suffer from overfitting the overcontrolled, undercontrolled, and resilient solution (Bohane et al., 2017).

Combining results from clustering analyses and transdiagnostic investigations, the goal of this study was to address these gaps by using a person-centered analytic technique on a sample of patients with varied clinical profiles, and healthy controls, subscribing to a fundamentally dimensional framework. We took a confirmatory approach in incorporating measures of overcontrol and undercontrol that have repeatedly been demonstrated to result in distinct personality profiles. However, drawing from both empirical studies investigating the personality traits impacting psychopathology (e.g., Koob and Volkow, 2010; Kotov et al., 2010; Judd et al., 2013; McEvoy et al., 2013; Paulus et al., 2015) as well as transdiagnostic and population-based profiling studies (e.g., Eaton et al., 2011; Rosellini and Brown, 2014) we incorporated an exploratory element by including additional personality trait measures that reflect negative affectivity and interpersonal dysregulation.

We aimed to test three hypotheses. Firstly, we expected 3–5 profiles resembling the overcontrolled, undercontrolled and low psychopathology classes to be distinguishable. Secondly, in addition to perfectionism and impulsivity, we anticipated somatic and psychic anxiety, stress susceptibility, mistrust, embitterment, irritability, and detachment to meaningfully contribute to profile differentiation. We assessed these hypotheses by latent profile analysis (LPA) with measures of these traits included as indicator variables. Additionally, after extracting the personality profiles, we validated their coherence by relating them to alternative instruments designed to measure similar constructs. Finally, we hypothesized profile membership to differentially determine variation in state anxiety, depression, emotion regulation difficulties and disordered eating, and evaluated this by relating profiles to distal outcome measures.

## 2. Materials and methods

### 2.1. Participants

The sample comprised 427 women (total sample, mean age *M* = 22.78, *SD* = 7.16), out of whom 249 (58.3%) had been diagnosed with an ED, 64 had a primary diagnosis of mood and anxiety disorders or SUDs (MOOD-SUD, 15.0%) and 114 (26.7%) were healthy controls. 63.6% of patients had at least one comorbid diagnosis. ED, MOOD-SUD participants, and healthy controls were matched on age and education.

Exclusion criteria for patients included intellectual disability, acute psychotic episodes, and involuntary hospitalization. Exclusion criteria for controls included current but not lifespan diagnoses of mental disorders.

Healthy controls and patients were profiled together based on a core assumption of dimensionality of investigated traits. Additionally, Schaefer et al. (2017) have shown only a minority in the population to not develop a psychiatric disorder over their life course, hinting at controls’ exclusion from explanatory models being unmerited.

Patients were recruited in an inpatient setting at the University of Tartu Clinic, the control sample *via* university mailing lists and public calls. Data were collected by trained clinical psychologists. Written informed consent from the participants was obtained after the nature of the procedures had been fully explained. Study design was reviewed and approved by the Research Ethics Committee of the University of Tartu (243/T-20, 196/T-17).

### 2.2. Measures

#### 2.2.1. LPA indicator measures

##### 2.2.1.1. Personality

Personality was profiled with the Swedish Universities Scales of Personality (SSP; Gustavsson et al., 2000; Aluoja et al., 2009). The SSP is a 91-item self-report questionnaire comprising 13 scales, seven items each (α = 0.58–0.85 scale level; Aluoja et al., 2009). Items are assessed on a 4-point scale. Composite scores of seven scales were used as indicators: somatic and psychic trait anxiety, stress susceptibility, detachment, embitterment, trait irritability and mistrust. Psychic and somatic anxiety and stress susceptibility have been shown to load onto a general negative affectivity factor, but while the two first scales reflect general proneness to anxiety, somatic anxiety is comparable to somatic anxiety in Rosellini and Brown (2014; Gustavsson et al., 2000; Aluoja et al., 2009). Trait irritability and embitterment reflect dysphoric mood and encapsulate facets of anxiety and aggression, while detachment encompasses withdrawal from social interactions and is significantly correlated with mistrust (Gustavsson et al., 2000; Aluoja et al., 2009). Aluoja et al. (2009) have shown detachment and mistrust to be strongly negatively correlated with extraversion on the Big Five personality scales, while Eaton et al. (2011) showed mistrust to contribute to personality profiles, lending support to the theory that higher scores on these interpersonal dysregulation measures could reflect a transdiagnostic vulnerability factor.

##### 2.2.1.2. Impulsivity

Impulsivity was assessed with Dickman’s Impulsivity Inventory (DII; Dickman, 1990). The DII is a 23-item (11 to tap functional impulsivity and 12 to assess dysfunctional impulsivity) self-report questionnaire (α = 0.74–0.85 scale level). Items are rated on a 5-point scale. In this study, scale scores were used.

##### 2.2.1.3. Perfectionism

Perfectionism was assessed with the Multidimensional Perfectionism Scale (MPS; Frost et al., 1990). The MPS has 35 items, making up four scales: concern over mistakes, parental standards and expectations, personal standards, and organization (total α = 0.90). Items are rated on a 5-point scale. Two composite scores—positive (personal standards and organization) and negative perfectionism (concern over mistakes and parental standards)—were included.

#### 2.2.2. Alternative instrument validation measures

##### 2.2.2.1. Personality

As alternative instruments to the SSP, four factor scores from the International Personality Item Pool NEO were used (EPIP-NEO, Goldberg, 1999, Mõttus et al., 2006). The 240-item self-report instrument assesses 30 different personality facets, and reflects five higher-order factors: neuroticism, extraversion, openness to experience, agreeableness, and conscientiousness (α = 0.89–0.95 factor level; Mõttus et al., 2006). We omitted openness to experience from this study due to weak associations with the SSP scales (Aluoja et al., 2009). In addition to reflecting personality traits measured by the SSP, we took conscientiousness to covary with and act as a proxy to perfectionism, especially in conjunction with high levels of neuroticism (see, e.g., Smith et al., 2019).

##### 2.2.2.2. Trait anxiety

As an alternative instrument to the SSP’s somatic and psychic anxiety and stress susceptibility, the State–Trait Anxiety Inventory (trait scale) was used (STAI; Spielberger et al., 1983). The STAI is a two-factor 40-item self-report questionnaire that measures state anxiety and general proneness to anxiety (α = 0.90–0.92 scale level). Items are rated on a 4-point scale.

##### 2.2.2.3. Impulsivity

As an alternative instrument to the DII, Barratt Impulsivity Scales’ total score was used (BIS-11; Patton et al., 1995). The BIS-11 is a 30-item self-report scale that has a six-factor primary level structure and a three-factor second order structure (total α = 0.83). Items are rated on a 4-point scale.

#### 2.2.3. Profile membership associations with distal outcomes

##### 2.2.3.1. Diagnosis

Diagnoses were established with the Mini International Neuropsychiatric Interview (M.I.N.I; Sheehan et al., 1998). The M.I.N.I is a brief structured clinician-administered interview. Test–retest reliability of the original instrument varies (0.52–1.00) across scales, interrater reliability ranges between 0.89 and 1.00.

##### 2.2.3.2. Emotion regulation difficulties

Emotion regulation was assessed with the total score of the Difficulties in Emotion Regulation Scale (DERS; Gratz and Roemer, 2004). The 36-item DERS reflects six factors: nonacceptance of emotional responses, difficulties engaging in goal-directed behaviors, impulse control difficulties, lack of emotional awareness, limited access to emotion regulation strategies, and lack of emotional clarity (total α = 0.93). Items are rated on a 5-point scale.

##### 2.2.3.3. Depression

Depression was assessed with the self-report Montgomery-Åsberg Depression Rating Scale (MADRS; Svanborg and Åsberg, 1994). The MADRS has 9 items, rated on a 7-point scale (α = 0.83; Fantino and Moore, 2009).

##### 2.2.3.4. State anxiety

State anxiety was assessed with the STAI state scale (Spielberger et al., 1983).

##### 2.2.3.5. Disordered eating

Disordered eating was assessed with the 29-item self-report measure Eating Disorder Assessment Scale (EDAS; Akkermann, 2010). Items are rated on a 6-point scale and form four subscales: restrained eating, binge eating, purging, and preoccupation with body image and body weight (α = 0.90–0.93 scale level). All scales were included.

### 2.3. Analytic strategy

To test the first and second hypotheses, profiles were extracted *via* LPA. LPA classifies individuals into latent profiles based on observed variation in continuous indicator variables (Muthén and Muthén, 1998–2012). The full information maximum likelihood method was applied to missing data in indicator variables to retain power and reduce bias (Cham et al., 2017). Consistently missing data patterns were excluded from analysis; validation analyses used case-by-case deletion.

The first step was to iteratively assess 1–8-profile models in the total sample. To correct for solutions converging on local maxima, starting value sets were increased to 1,000 initial and 250 final stage optimizations. A maximum of 20 initial stage iterations were run. The best-fitting model was chosen based on parsimony, theoretical interpretability, and goodness-of-fit indicators: logarithm of the likelihood of fit (LL), Bayesian Information Criterion (BIC), sample-adjusted BIC (SABIC), and Akaike’s Information Criterion (AIC; Tein et al., 2013; Ferguson et al., 2020). Lower absolute values of LL, BIC, SABIC, and AIC demonstrate better fit, entropy values surpassing 0.80 indicate minimal uncertainty and significant BIC, SABIC, and AIC postulate superiority of k versus k-1 profiles (Masyn, 2013). Based on previous research, this study primarily focused on agreement of BLRT and LMR-LRT with BIC (Nylund et al., 2007; Morgan, 2015).

To evaluate the quality of the best-fitting model, profile means were compared across instruments that assess constructs resembling indicator variables. To explore the third hypothesis, associations with distal outcomes were assessed by comparisons of outcome measure means based on categorical profile membership. BCH tests are comparatively reported in Supplementary materials, since BCH independently has been shown to be unreliable when outcome measures are non-normally distributed (Asparouhov and Muthén, 2014). The omega squared statistic was used to calculate effect sizes for analyses of variance (ANOVAS) to reduce bias (rule of thumb 0.01 small, 0.06 medium, and 0.14 large effect size; Field, 2013) and Cramér’s V for cross-tabulations (rule of thumb 0.05 small, 0.15 medium, and 0.25 large effect size; Cohen, 1988). See Supplementary materials (S1) for more on methodological choices.

LPAs were conducted in Mplus (version 7.4; Muthén and Muthén, 1998–2012), additional analyses in SPSS 26.0. Data was visualized using R (version 4.1.1; R Core Team, 2021) and the ggplot2 package (version 3.3.5; Wickham, 2016).

## 3. Results

### 3.1. Descriptive statistics

Data from seven subjects were consistently missing and omitted from LPAs. Descriptive statistics for all variables are presented in Supplementary Table 1 (S2).

### 3.2. Latent profile analysis

#### 3.2.1. Model Fit estimation

Summary model fit statistics for the total sample are reported in Table 1. Fit indices for the sample without controls as well as subsamples are presented in Supplementary materials (S3), since independently, they lack statistical power.

**Table 1:** Fit indices for 1–8 profile latent profile analyses in the total sample.

No	LL	AIC	BIC	SABIC	Entropy	SMALL %	LMR (*p*)	BLRT (*p*)
1	−16,633.62	33,311.23	33,400.12	33,330.31	—	—	—	—
2	−16,062.65	32,193.29	32,330.66	32,222.77	0.84	49.5%	0.003	<0.001
3	−15,830.15	31,752.30	31,938.15	31,792.18	0.87	20.5%	<0.001	<0.001
4	−15,768.51	31,653.03	31,887.36	31,703.31	0.81	13.8%	0.47	<0.001
5	**−15,724.51**	**31,589.03**	**31,871.85**	**31,649.71**	**0.80**	**13.3%**	**0.23**	**<0.001**
6	−15,693.33	31,550.66	31,881.96	31,621.75	0.81	5.2%	0.46	<0.001
7	−15,664.56	31,517.11	31,896.89	31,598.60	0.82	2.6%	0.34	<0.001
8	−15,639.70	31,491.39	31,919.66	31,583.29	0.80	2.4%	0.69	<0.001

Best-fitting model in bold. No = number of profiles in model; LL = log likelihood; AIC = Akaike’s Information Criterion; BIC = Bayesian Information Criterion, SABIC = sample-adjusted Bayesian information criterion; SMALL % = proportion of sample in smallest profile; LMR = adjusted Lo–Mendell–Rubin likelihood ratio test; BLRT = bootstrap likelihood ratio test.

Total sample (*N* = 420) LPAs provided mixed evidence for the 3- and 5-profile solutions. Entropy of 0.87 served as evidence in favor of the 3-profile solution, LMR-LRT indicated the model’s superiority in comparison to the 2-profile model. The 5-profile model was supported by the best BIC value and better LL, AIC and SABIC values in comparison to the 3-profile model. Since entropy values surpassing 0.80 are taken to reflect good fit, and the adjusted LMR-LRT can underpredict profile number, the 5-profile model garnered more support in total and was thus selected as the best model.

#### 3.2.2. Profile characteristics

3–5-profile solutions were studied to analyze interpretability and reasons for divergent evidence in model detection.

**Figure 1:**
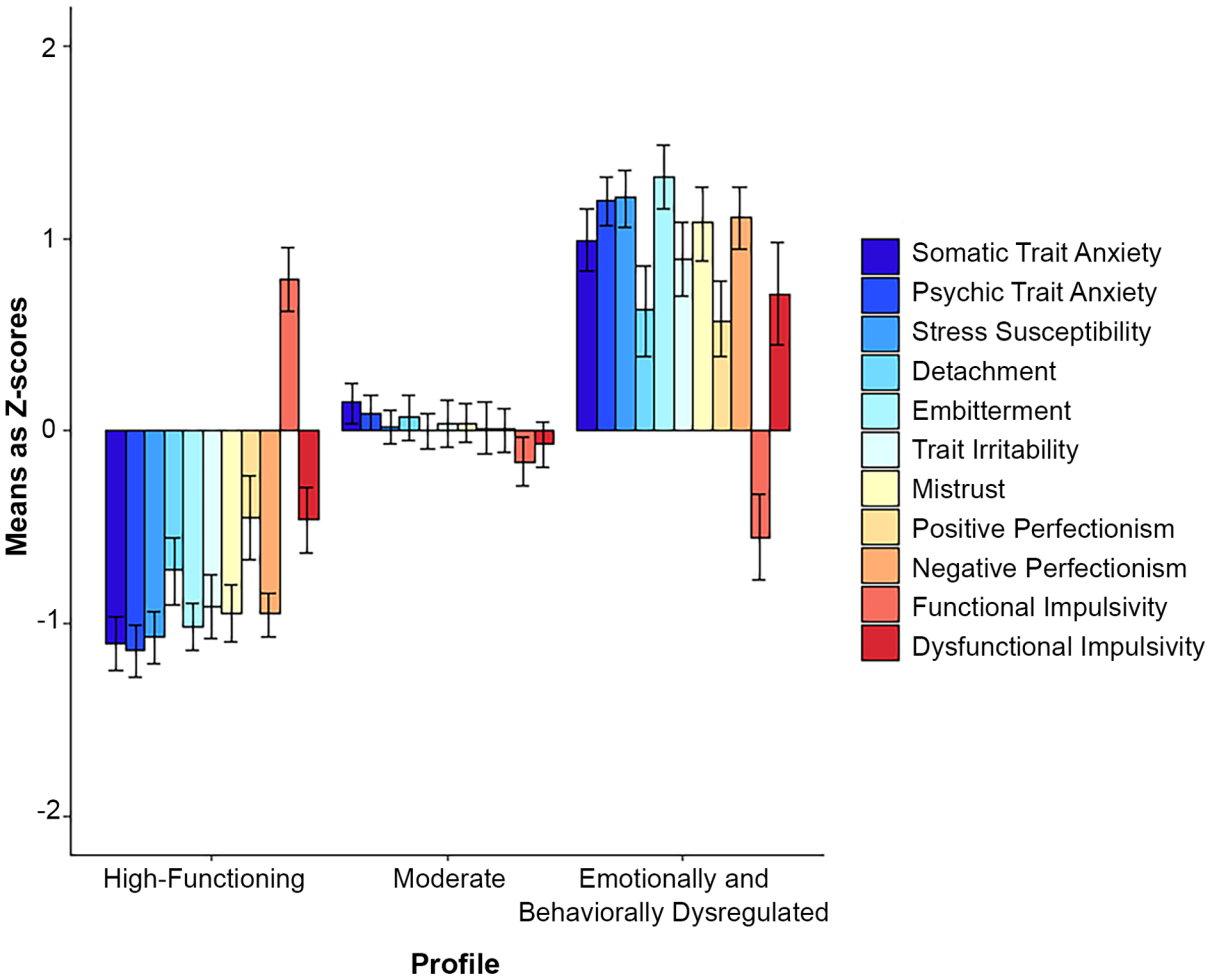
Z-scores with 95% confidence intervals in the 3-profile total sample solution.

In the 3-profile model (Figure 1), the following profiles emerged: (1) a profile characterized by low psychic and somatic anxiety, stress susceptibility, embitterment, detachment, irritability and mistrust, as well as low perfectionism and dysfunctional impulsivity and high functional impulsivity—the high-functioning profile; (2) a profile with average scores on all indicator variables—the moderate profile; (3) a profile with very high somatic and trait anxiety, stress susceptibility, embitterment, mistrust and perfectionism; high dysfunctional impulsivity, irritability, and low functional impulsivity—the emotionally and behaviorally dysregulated profile.

**Figure 2:**
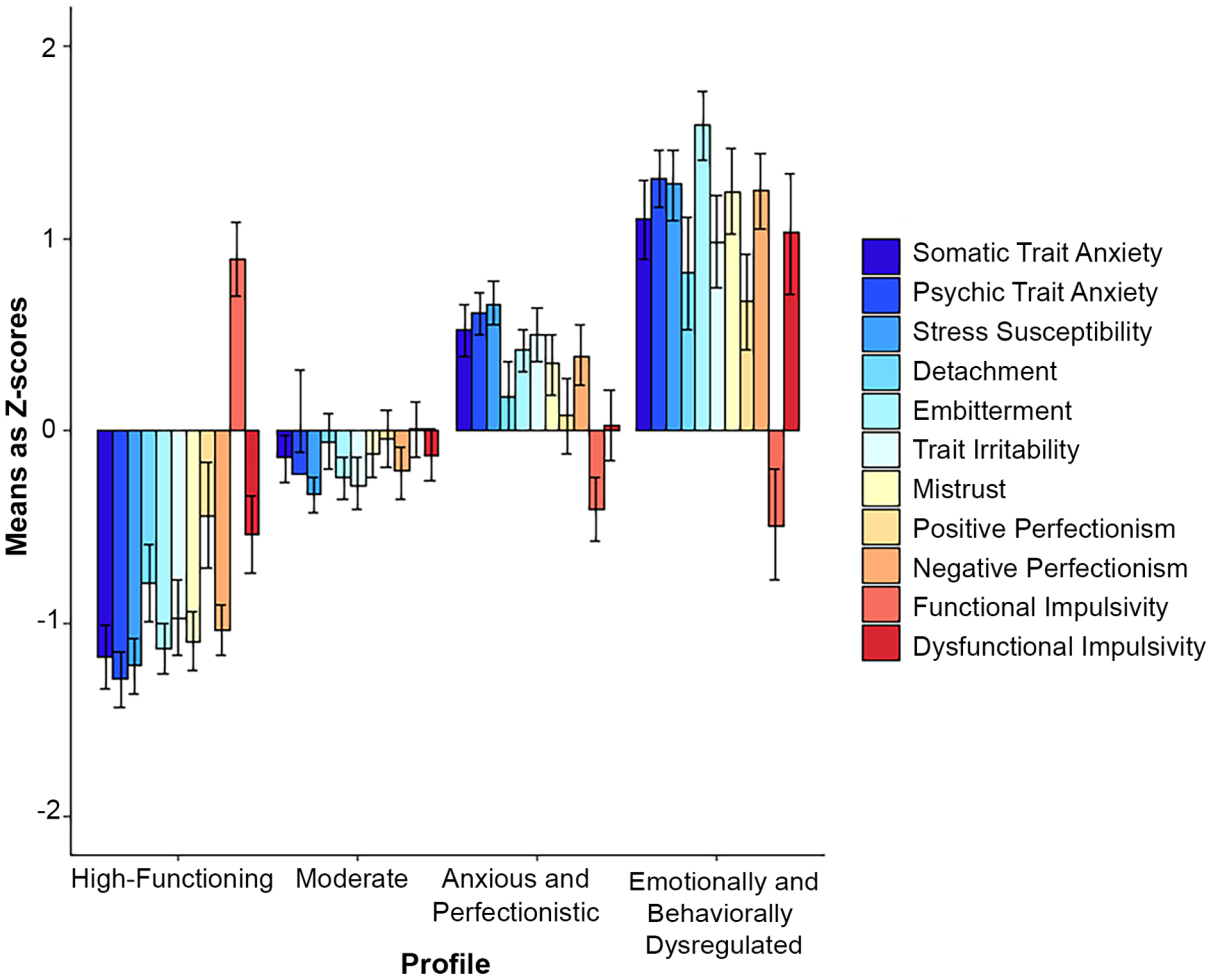
Z-scores with 95% confidence intervals in the 4-profile total sample solution.

In the 4-profile model (Figure 2), the moderate profile separated into two, leaving one moderate and one anxious and perfectionistic profile, with highest scores on the somatic and psychic anxiety and stress susceptibility scales, pronounced negative perfectionism and elevated embitterment and irritability.

**Figure 3:**
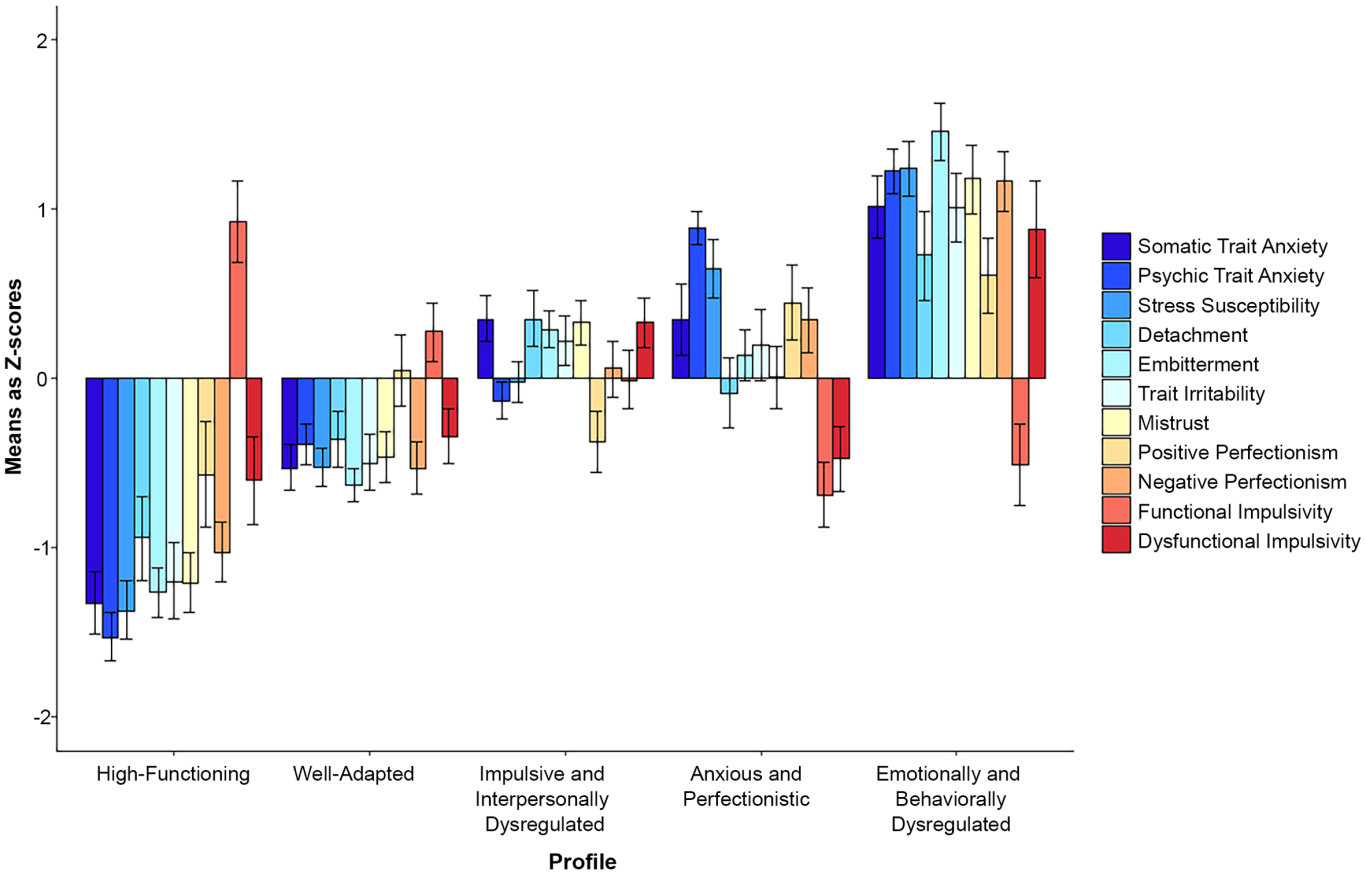
Z-scores with 95% confidence intervals in the 5-profile total sample solution.

With the extraction of an additional, fifth profile (Figure 3), the moderate profile developed into a well-adapted profile (*n* = 112) differing from the high-functioning (*n* = 56) profile only quantitatively. Two separate profiles emerged from the anxious and perfectionistic profile: an impulsive and interpersonally dysregulated profile, characterized by elevated impulsivity, embitterment, irritability, mistrust, and somatic anxiety (*n* = 114), and an anxious and perfectionistic profile (*n* = 66), characterized by high perfectionism, psychic anxiety, and stress susceptibility. The emotionally and behaviorally dysregulated profile remained intact (*n* = 72). Across profiles, comparisons of indicator variable means were statistically significant, results are presented in Supplementary materials (S4). Graphical presentations of the five-profile model in our subsamples are also available in Supplementary materials (S5).

### 3.3. Validation analyses of profile extraction

Classification certainty in the 5-profile solution was captured by entropy of 0.80. Probabilities for the most likely latent profile ranged between 0.81 and 0.93, the mean likelihood of individuals to belong to the second-best profile ranged between 0.03 and 0.11.

Alternative instrument validation analysis results are presented in Table 2, BCH results in Supplementary materials (S4). The high-functioning profile was characterized by the highest extraversion and lowest neuroticism. The well-adapted participants were generally comparable, yet exhibited more trait anxiety and neuroticism compared to the first profile. The impulsive and interpersonally dysregulated profile demonstrated pronounced impulsivity and low conscientiousness in comparison to the two well-functioning classes as well as the anxious and perfectionistic profile. Additionally, these participants were relatively low in agreeableness. While the anxious and perfectionistic group had higher levels of neuroticism and lower extraversion compared to the impulsive and interpersonally dysregulated people, they also exhibited more conscientiousness, high agreeability and low impulsivity. The emotionally and behaviorally dysregulated profiles had the highest impulsivity, anxiety and neuroticism, and lowest scores on conscientiousness, agreeableness and extraversion measures.

**Table 2:** Comparison of means of alternative instruments across profiles in the best-fitting total sample 5-profile model.

Variable	Profile					ANOVA and Kruskal-Wallis H				
	High-functioning (*n* = 56)	Well-adapted (*n* = 112)	Impulsive and interpersonally dysregulated (*n* = 114)	Anxious and perfectionistic (*n* = 66)	Emotionally and behaviorally dysregulated (*n* = 72)	*df2*	*F*	*H*(4)	ω^2^	95% CI for ω^2^
	*M*(*SD*)	*M*(*SD*)	*M*(*SD*)	*M*(*SD*)	*M*(*SD*)					
Impulsivity	53.2 (9.1)^c^^,^^e^	54.3 (10.8)^c^^,^^e^	63.4 (9.8)^a^^,^^b^^,^^d^	57.8 (9.8)^c^^,^^e^	67.7 (12.8)^a^^,^^b^^,^^d^	270	17.32*	55.76*	0.13	[0.07,0.19]
Trait anxiety	33.0 (9.6)^b^^,^^c^^,^^d^^,^^e^	41.1 (11.7)^a^^,^^c^^,^^d^^,^^e^	53.3 (11.2)^a^^,^^b^^,^^e^	58.5 (9.5)^a^^,^^b^	63.9 (11.1)^a^^,^^b^^,^^c^	273	52.40*	117.88*	0.41	[0.35,0.48]
Conscientiousness	142.4 (19.9)^c^^,^^e^	135.1 (18.6)^c^^,^^e^	115.7 (21.7)^a^^,^ ^b^	127.3 (18.0)^e^	103.1 (27.1)^a^^,^^b^^,^^d^	128	13.47*	35.92*	0.23	[0.13,0.33]
Neuroticism	54.5 (24.8)^b^^,^^c^^,^^d^^,^^e^	78.0 (18.2)^a^^,^^c^^,^^d^^,^^e^	95.9 (17.3)^a^^,^^b^^,^^d^^,^^e^	112.4 (22.1)^a^^,^^b^^,^^c^^,^^e^	139.9 (16.1)^a^^,^^b^^,^^c^^,^^d^	128	62.73*	85.19*	0.65	[0.58,0.71]
Agreeableness	142.6 (10.6)^c^^,^^e^	135.0 (12.2)^c^^,^^d^^,^^e^	122.5 (20.5)^a^^,^^b^^,^^d^	144.9 (11.5)^b^^,^^c^^,^^e^	114.3 (27.6)^a^^,^^b^^,^^d^	53	10.69^f^^,^*	35.29*	0.40	[0.21,0.52]
Extraversion	122.2 (20.3)^d^^,^^e^	119.0 (20.1)^d^^,^^e^	109.7 (20.9)^e^	97.8 (26.0)^a^^,^^b^	91.6 (27.2)^a^^,^^b^^,^^c^	128	8.16*	25.42*	0.19	[0.10,0.29]

Tukey test applied, *post-hoc* tests significant at *p* < 0.05. For all ANOVA tests, *df1* = 4. Effect sizes calculated for parametric tests.

a Statistically significantly different from high-functioning profile.

b Different from well-adapted profile.

c Different from impulsive and interpersonally dysregulated profile.

d Different from anxious and perfectionistic profile.

e Different from emotionally and behaviorally dysregulated profile.

f Welch ANOVA, Games-Howell test applied, effect size is an estimation.

* *p* < 0.001.

### 3.4. Profiles as predictors of diagnoses and emotional state outcomes

Comparisons of distal outcomes are presented in Table 3 (BCH in S4). The high-functioning and well-adapted profile demonstrated the lowest levels of depression, state anxiety and eating pathology and differences between the two profiles were quantitative in nature. Among the three dysregulated profiles, disturbances generally continuously increased, moving from the impulsive and interpersonally dysregulated to the anxious-perfectionistic to the emotionally and behaviorally dysregulated participants. Differential associations arose with emotion regulation difficulties, bingeing and purging: the anxious-perfectionistic group demonstrated less of these symptoms in comparison to the impulsive participants.

**Table 3:** Comparison of means of distal outcomes across profiles in the best-fitting total sample 5-profile model.

Variable	Profile					ANOVA and Kruskal-Wallis H				
	High-functioning (*n* = 56)	Well-adapted (*n* = 112)	Impulsive and interpersonally dysregulated (*n* = 114)	Anxious and perfectionistic (*n* = 66)	Emotionally and behaviorally dysregulated (*n* = 72)	*df2*	*F*	*H*(4)	ω^2^	95% CI for ω^2^
	*M*(*SD*)	*M*(*SD*)	*M*(*SD*)	*M*(*SD*)	*M*(*SD*)					
Depression	5.7 (5.3)^b^^,^^c^^,^^d^^,^^e^	12.4 (9.5)^a^^,^^c^^,^^d^^,^^e^	18.1 (9.3)^a^^,^^b^^,^^e^	20.3 (8.8)^a^^,^^b^^,^^e^	27.5 (10.0)^a^^,^^b^^,^^c^^,^^d^	126	52.46^f^^,^*	103.46*	0.61	[0.52, 0.67]
State anxiety	30.5 (10.8)^b^^,^^c^^,^^d^^,^^e^	39.9 (15.0)^a^^,^^c^^,^^d^^,^^e^	47.2 (13.2)^a^^,^^b^^,^^e^	50.7 (13.1)^a^^,^^b^^,^^e^	58.5 (13.6)^a^^,^^b^^,^^c^^,^^d^	121	31.68^f^^,^*	80.74*	0.49	[0.38, 0.57]
Emotion regulation	30.5 (22.9)^b^^,^^c^^,^^d^^,^^e^	58.7 (25.1)^a^^,^^c^^,^^d^^,^^e^	85.6 (23.2)^a^^,^^b^^,^^e^	58.5 (13.6)^a^^,^^b^^,^^e^	112.3 (20.9)^a^^,^^b^^,^^c^^,^^d^	160	54.28*	97.85*	0.56	[0.48, 0.63]
Restrained eating	10.7 (8.1)^b^^,^^c^^,^^d^^,^^e^	16.0 (9.9)^a^^,^^d^^,^^e^	17.2 (10.2)^a^^,^^d^^,^^e^	22.8 (10.4)^a^^,^^b^^,^^c^	23.9 (9.4)^a^^,^^b^^,^^c^	185	19.76^f^^,^*	67.53*	0.32	[0.22, 0.39]
Binge eating	8.2 (7.3)^b^^,^^c^^,^^d^^,^^e^	13.6 (8.8)^a^^,^^c^^,^^d^^,^^e^	18.6 (9.5)^a^^,^^b^	17.8 (10.7)^a^^,^^b^	20.3 (12.3)^a^^,^^b^	182	16.69^f^^,^*	64.97*	0.30	[0.20, 0.38]
Purging	1.5 (3.9)^c^^,^^d^^,^^e^	2.9 (5.4)^c^^,^^e^	5.4 (6.5)^a^^,^^b^	5.1 (5.8)^a^	8.0 (7.0)^a^^,^^b^	186	12.88^f^^,^*	66.13*	0.22	[0.13, 0.30]
Preoccupation with weight	9.0 (9.3)^b^^,^^c^^,^^d^^,^^e^	13.6 (10.4)^a^^,^^c^^,^^d^^,^^e^	19.3 (11.2)^a^^,^^b^^,^^d^^,^^e^	25.0 (10.9)^a^^,^^b^^,^^c^	28.6 (9.6)^a^^,^^b^^,^^c^	184	40.66^f^^,^*	120.69*	0.49	[0.40, 0.55]

Tukey test applied, *post-hoc* tests significant at *p* < 0.05. For all ANOVA tests, *df1* = 4. Effect sizes calculated for parametric tests.

a Statistically significantly different from high-functioning profile.

b Different from well-adapted profile.

c Different from impulsive and interpersonally dysregulated profile.

d Different from anxious and perfectionistic profile.

e Different from emotionally and behaviorally dysregulated profile.

f Welch ANOVA, Games-Howell test applied, effect size is estimation.

* *p* < 0.001.

On average, individuals in the high-functioning profile presented with *M* = 0.6 (*SD* = 0.9) comorbid disorders, the well-adapted profile *M* = 1.2 (*SD* = 1.6), impulsive and interpersonally dysregulated *M* = 2.0 (*SD* = 1.8), anxious and perfectionistic *M* = 2.5 (*SD* = 1.7) and emotionally and behaviorally dysregulated profile *M* = 3.7 (*SD* = 2.5; Welch corrected *F* (4, 188) = 35.76, *p* < 0.001; ω^2^ = 0.42, 95% CI [0.33, 0.49]; non-parametric *H*(4) = 105.74, *p* < 0.001). Games-Howell post-hoc comparisons were significant at *p* < 0.05 for all profile comparisons, except between the impulsive and interpersonally dysregulated and the anxious and perfectionistic profile. Prevalence of ICD-10 disorders is displayed in Table 4. Most participants (62.5%) in the high-functioning profile and nearly half (42.9%) of the individuals labeled as well-adapted were healthy controls. The impulsive and interpersonally dysregulated class had a high proportion of patients with BN (35.1%) and depression (36.8%), while the anxious-perfectionistic profile was mostly made up by AN (39.4%), BN (31.8%) and GAD (28.8%) patients. In comparison, the emotionally and behaviorally dysregulated class exhibited more SUDs (26.4%), PTSD (20.8%) and anxiety disorders (GAD 37.5%, social phobia 30.6%, agoraphobia 27.8%).

**Table 4:** Prevalence of disorders across profiles in the best-fitting total sample 5-profile model.

Disorder	*n* (% of *N* = 420)	Profile					χ^2^(4)	Cramér’s *V*	95% CI for *V*
		High-functioning (*n* = 56)	Well-adapted (*n* = 112)	Impulsive and interpersonally dysregulated (*n* = 114)	Anxious and perfectionistic (*n* = 66)	Emotionally and behaviorally dysregulated (*n* = 72)			
*Anorexia nervosa*	119 (28.3%)	16 (28.6%)	27 (24.1%)	25 (21.9%)	26 (39.4%)	25 (34.7%)	8.71	0.14	[0.09, 0.27]
*Bulimia nervosa*	108 (25.7%)	3 (5.4%)^c^^,^^d^^,^^e^	18 (14.8%)^c^^,^^e^	40 (35.1%)^a^^,^^b^	21 (31.8%)^a^	26 (36.1%)^a^^,^^b^	28.21***	0.26	[0.20, 0.35]
BED	18 (4.0%)	1 (1.8%)^d^	2 (1.8%)	3 (2.6%)	8 (12.1%)^a^	4 (4.2%)	13.88**	0.18	[0.08, 0.32]
SUD	53 (12.6%)	1 (1.8%)^e^	9 (8.0%)^e^	15 (13.2%)	9 (13.6%)	19 (26.4%)^a^^,^^b^	20.57***	0.22	[0.15, 0.33]
Depression	153 (36.4%)	5 (8.9%)^c^^,^^d^^,^^e^	26 (23.2%)^d^^,^^e^	42 (36.8%)^a^^,^^e^	34 (51.5%)^a^^,^^b^	46 (63.9%)^a^^,^^b^^,^^c^	55.44***	0.37	[0.30, 0.45]
Bipolar disorder	12 (2.9%)	0 (0%)	1 (0.9%)	4 (3.5%)	2 (3.0%)	5 (6.9%)	7.58	0.14	[0.09, 0.25]
GAD	78 (18.6%)	3 (5.4%)^d^^,^^e^	11 (9.8%)^d^^,^^e^	18 (15.8%)^e^	19 (28.8%)^a^^,^^b^	27 (37.5%)^a^^,^^b^^,^^c^	33.53***	0.28	[0.20, 0.38]
Social phobia	64 (15.2%)	1 (1.8%)^d^^,^^e^	9 (8.0%)^e^	18 (15.8%)	14 (21.2%)^a^	22 (30.6%)^a^^,^^b^	26.69***	0.25	[0.18, 0.35]
Agoraphobia	50 (11.9%)	1 (1.8%)^e^	5 (4.5%)^e^	16 (14.0%)	8 (12.1%)	20 (27.8%)^a^^,^^b^	28.64***	0.26	[0.18, 0.38]
Panic disorder	29 (6.9%)	0 (0%)^e^	1 (0.9%)^e^	9 (7.9%)	4 (6.1%)	15 (20.8%)^a^^,^^b^	31.95***	0.28	[0.19, 0.39]
PTSD	23 (5.5%)	0 (0%)^e^	4 (3.6%)^e^	2 (1.8%)^e^	2 (3.0%)^e^	15 (20.8%)^a^^,^^b^^,^^c^^,^^d^	40.13***	0.31	[0.19, 0.43]
OCD	23 (5.5%)	0 (0%)	3 (2.7%)	10 (8.8%)	2 (3.0%)	8 (11.1%)	12.36*	0.17	[0.11, 0.27]
Controls	114 (27.1%)	35 (62.5%)^c^^,^^d^^,^^e^	48 (42.9%)^c^^,^^d^^,^^e^	24 (21.1%)^a^^,^^b^^,^^e^	5 (7.6%)^a^^,^^b^	2 (2.78%)^a^^,^^b^^,^^c^	85.92***	0.45	[0.38, 0.54]

Number of diagnoses and, in parentheses, proportion of profile. Bonferroni test applied, post-hoc tests significant at *p* < 0.05. BED = binge eating disorder; SUD = substance use disorder; depression = includes dysthymia, bipolar disorder = includes mania; GAD = generalized anxiety disorder; PTSD = post-traumatic stress disorder; OCD = obsessive–compulsive disorder.

a Statistically significantly different proportion of cases than high-functioning profile.

b Different proportion of cases than well-adapted profile.

c Different proportion of cases than impulsive and interpersonally dysregulated profile.

d Different proportion of cases than anxious and perfectionistic profile.

e Different proportion of cases than the emotionally and behaviorally dysregulated profile.

* *p* < 0.05.

** *p* < 0.01.

*** *p* < 0.001.

## 4. Discussion

The results of this study underscore the importance of testing personality-based profiling models on varied samples. With five distinct and clinically significant profiles emerging, we found support for all three hypotheses. Firstly, five profiles were extracted. These were labelled the high-functioning, the well-adapted, the impulsive and interpersonally dysregulated, the anxious and perfectionistic and the emotionally and behaviorally dysregulated profiles. The detected profiles mirrored the overcontrolled (anxious and perfectionistic), undercontrolled (dysregulated profiles), and low psychopathology classes (high-functioning and well-adapted). Secondly, all indicators differed significantly across profiles, demonstrating the benefits of utilizing additional measures. Finally, class membership was found to predict current emotional state with anxiety, depression, disordered eating, and difficulties in emotion regulation differing across profiles.

### 4.1. Profile identification

Our findings converge with previous studies, especially with research on EDs (Bohane et al., 2017). Important similarities with Rosellini and Brown (2014), who incorporated a diverse clinical sample and more measures, emerged. Severe comorbid (here emotionally and behaviorally dysregulated) and negligible-mild (high-functioning and well-adapted) classes were postulated in both studies, the anxious and perfectionistic profile in the current study appears to encapsulate facets of the mildly-neurotic and obsessed-worried classes in Rosellini and Brown (2014), while the impulsive and interpersonally dysregulated profile reflects a combination of the social-depressed and panic-somatic classes. We validated our profiles by relating them to traits from the Five Factor model. Findings from this post-LPA validation also align with previous research – our undercontrolled groups demonstrated low agreeableness and conscientiousness and our overcontrolled profile had high neuroticism and conscientiousness (for a review, see Yin et al., 2021). Such agreement of results points towards a common trait-based disposition to develop and maintain psychopathology among affective disorders and affirms the generalizability of results from less diverse samples.

While the emergence of high-functioning and combined perfectionism-impulsivity classes has been well documented, the precise number of meaningful profiles remains controversial. Differences in the number of extracted classes (e.g., Boone et al., 2014) and characteristics of the profiles can result from our utilization of additional LPA indicator measures, including scales for assessing interpersonal dysregulation (Spinhoven et al., 2012; Christian et al., 2021).

### 4.2. Profile characteristics

Elucidating the nature of the profiles, we found support for the claim that personality traits can be protective factors in people with EDs, SUDs and mood and anxiety disorders. High-functioning and well-adapted participants displayed low comorbidity and limited emotional disturbance, aligning with the finding that membership in resilient classes consistently predicts better treatment response (Wardenaar et al., 2014).

Similarly to Yin et al. (2021), we found neuroticism and proxies of general negative affectivity, like somatic and psychic trait anxiety, to show the largest effect sizes in differentiating between the profiles. Interestingly, we found the classes with average trait-and state-level disturbance to be differentiated by varied patterns of interpersonal dysfunction and facets of anxiety. This highlights the necessity of nuanced profile extraction, including distinguishing between other-directed facets of negative affectivity and diverse presentations of anxiety (measured by stress susceptibility, psychic and somatic trait anxiety; Hartmann et al., 2010; Judd et al., 2013). As such, our results indirectly affirm the relevance of antagonistic, peculiar, and disinhibited traits as contributors to personality-based profiles and demonstrate how pathways of negative affectivity are mediated by different maintenance factors (Krueger and Eaton, 2010; Paulus et al., 2015).

Our study replicated the finding that the emotionally and behaviorally dysregulated profile displays the most psychopathology and state disturbances, aligning with Boone et al. (2014), Christian et al. (2021) and Rosellini and Brown (2014). Additionally, this profile exhibited the highest rates of comorbidity. These results could be interpreted as demonstrating the mediating role of emotion regulation in the interplay of state and trait features (Abdi and Pak, 2019). One potential explanatory mechanism is that people in this profile perceive impulsive behavior as unwanted and perfectionistic tendencies serve as compensatory mechanisms (Boone et al., 2014). This view also suggests that a high base rate of negative affectivity and anxiety, often manifest in maladaptive perfectionism, can lead to impulsive action in response to negative emotions (Beauchaine and Zisner, 2017; Kim and Hodgins, 2018).

Profiles’ differential prediction of mental disorder diagnoses was also meaningful. While our results generally affirmed the findings relating overcontrolled profiles to internalizing psychopathology (e.g., AN, GAD) and undercontrolled groups to externalizing features (e.g., SUDs, BN), we believe these findings to be preliminary due to the disproportionate prevalence of different disorders in our sample (see also Kotov et al., 2017 for discussion on the internalizing and externalizing spectra).

### 4.3. A dimensional transdiagnostic account of affective disorders and EDs

Our results indicate that there is initial evidence in favor of personality-based classification and treatment of psychopathology. Firstly, applying a profiling approach to a sample with varied disorder presentations allowed us to reject the implications of current diagnostic borders. In our study, no profile contained only one diagnosis and it was not the case that all individuals with one diagnosis clustered in the same profile, hinting at within-disorder heterogeneity and cross-disorder homogeneity. Secondly, modeling the overcontrol-undercontrol relations on a mixed sample of patients and healthy controls and finding profile configurations similar to those previously reported in less diverse samples serves as evidence in favor of traits varying dimensionally across the population.

Such dimensional conceptualization can help elucidate the mechanisms *via* which the overlapping biopsychosocial basis of disorders results in comorbidity. For example, high prevalence of GAD, EDs and depression in the anxious and perfectionistic profile could hint at anxiety sensitivity being a transdiagnostic factor influencing symptomatology and requiring attention in treatment (Barlow et al., 2011; Norr et al., 2014). Since personality-based profiles differed across state-level disturbance, early identification of risk traits could help buffer the vulnerability to develop mental disorders (Hopwood et al., 2020). As such, our study underscores the potential of person-centered latent modelling techniques in clinical applications and highlights the necessity to go beyond state-level symptom-based profiling (Yarrington et al., 2022). Additionally, detection of co-varying impulsivity and perfectionism, especially accompanied by high levels of interpersonal dysregulation, should guide treatment choices to explicitly target maladaptive cognitions and behaviors deriving from these traits.

HiTOP promotes a similar transdiagnostic approach to pathology (Kotov et al., 2017). In our study, traits assigned to HiTOP spectra combined to make up the extracted profiles, rather than fell into discrete classes. For example, while the impulsive and interpersonally dysregulated profile reflected pathology more characteristic to the externalizing and detachment spectra, individuals also exhibited heightened somatic anxiety, and while the emotionally and behaviorally dysregulated profile was impulsive, this profile was also characterized by internalizing traits. Such results do not contradict HiTOP, yet they highlight the need for further investigation of between-spectra associations.

### 4.4. Limitations and future directions

This study has several limitations. Firstly, EDs were overrepresented in our sample. To account for potential bias, subsamples were profiled separately, revealing generally equivalent results, yet less pronounced impulsive profiles. Diagnostic distribution across profiles showed that non-ED diagnoses did not discretely group together, further proving that the model is not ED-specific. Regardless, results from small subsamples should be extrapolated with caution. Secondly, both personality and emotional state were assessed using self-report measures. Due to potential response bias and an assumption of responder insight, a multimethod assessment approach is merited in future investigations (Ganellen, 2007). Additionally, since our participants’ demographic profile was relatively homogenous and did not include men, results might not be applicable to a wider clinical population.

Despite these limitations, our results affirm the relevance of personality profiles in constructing both descriptive and explanatory accounts of pathology and highlight the importance of reverting to a dimensional symptom space. With this study’s differentiation between state and trait variables, comparative inclusion of 3–5-profile models and validation analyses, it serves as robust preliminary evidence of the extracted profiles and their clinical relevance.

We suggest three strands of future research to increase results’ applicability in clinical practice. Firstly, resilient, overcontrolled, and undercontrolled personality types have been shown to emerge in early childhood and be significant predictors of later functioning (e.g., Gilbert et al., 2021). Based on this, the profiles extracted in this study should be assessed longitudinally to examine temporal stability, especially in comparison with current nosologies. Secondly, profiles should be related to biological markers of pathology to determine their potential significance in establishing endophenotypes. Finally, further studies to assess whether profile membership predicts treatment response are warranted. Such research could help evaluate the relevance of the diathesis-stress model in treating personality profiles as risk factors and contribute to personalized interventions.

## Data availability statement

The data supporting the conclusions of this article will be made available by the authors upon request and in line with participants’ informed consent.

## Ethics statement

The studies involving human participants were reviewed and approved by the Research Ethics Committee of the University of Tartu. The patients/participants provided their written informed consent to participate in this study.

## Author contributions

HLS was involved in conceptualization, data curation and analysis, investigation, methodology, and writing-original draft. KA was involved in conceptualization, data curation, formal analysis, investigation, methodology, project administration, resources, supervision, and writing-review and editing. All authors contributed to the article and approved the submitted version.

## Acknowledgments

We would like to thank Sheryl Võsu, Kärol Soidla, Kerttu Petenberg and Elis Paasik for their contribution to data collection.

## Conflict of interest

The authors declare that the research was conducted in the absence of any commercial or financial relationships that could be construed as a potential conflict of interest.

## Publisher’s note

All claims expressed in this article are solely those of the authors and do not necessarily represent those of their affiliated organizations, or those of the publisher, the editors and the reviewers. Any product that may be evaluated in this article, or claim that may be made by its manufacturer, is not guaranteed or endorsed by the publisher.

## Supplementary material

SUPPLEMENTARY TABLE 1 S1_Data_Analysis_Procedure

SUPPLEMENTARY TABLE 2 S2_Descriptive_Statistics

SUPPLEMENTARY TABLE 3 S3_Model_Fit_Statistics

SUPPLEMENTARY TABLE 4 S4_Five-Profile_Model_Statistics

SUPPLEMENTARY TABLE 5 S5_Subsample_Profiles_Graphically
