# Supplementary material for: Bottom-up transdiagnostic personality subtypes are associated with state psychopathology: A latent profile analysis
Source: Front Psychol. 2023 Feb 21;14:1043394. doi: 10.3389/fpsyg.2023.1043394 (PMC9990091; doi:10.3389/fpsyg.2023.1043394)
Supplement: SUPPLEMENTARY TABLE 1 — S1_Data_Analysis_Procedure [file Table_1.docx]

Supplementary Material S1

Data Analysis Procedure

# **Choice of Starting Value Sets**

All LPA analyses were run with three sets of starting values to correct for converging on local maxima: random values, 100 initial and 20 final, and 1000 initial and 250 final stage optimizations, with final optimizations equal to or less than one quarter of initial values (Muthén and Muthén, 1998–2012). Only analyses with 1000 initial and 250 final stage optimizations are reported. Obtained results were compared in terms of starting value sets changing the order of k-class models in terms of fit and to test if bootstrap tests become or cease to be significant.

# Choice of Best-Fitting Model

Since comparative analyses have found BIC to outperform other information criteria indices and BIC is the most suitable indicator for analysis with many indicators, it was considered the preferential measure in this study, however, it can underpredict in smaller samples (Morgan, 2015). In this study, all indices were plotted to detect theoretically meaningful changes in values across models (Ferguson et al., 2020). Entropy was used to capture classification quality; values surpassing .80 indicate minimal uncertainty (Masyn, 2013). Finally, the adjusted Lo, Mendell and Rubin (LMR-LRT) and bootstrap likelihood ratio tests (BLRT) were used to assess extraction of k versus k–1 profiles to achieve optimal parsimony (Ferguson et al., 2020). For the LMR-LRT and BLRT tests, random starting values were set in two stages (2 1 100 20; for the k and k–1 profiles); in the BLRT test, the number of bootstrap sample draws was set to a maximum of 20. Simulation studies have shown BLRT to never reach a non-significance, so this study primarily focused on agreement of BLRT and LMR-LRT with BIC (Morgan, 2015; Masyn, 2013).

A best-fitting model was only chosen in the total sample, subsample statistics are reported for context and to allow for assessment of fit of the model with the number of classes established in total sample.

# Assumption of Conditional Independence

LPA assumes conditional independence, meaning that any covariation among indicator variables should be accounted for by the extracted latent variables (Lee et al., 2020). With all indicator variables continuous, testing for violations of conditional independence was hindered. The indicator variables in this study were significantly intercorrelated yet were intended to all measure distinct facets of the analyzed concepts, rendering an approach where indicator variables are allowed to load onto a continuous latent variable, unsuitable (Lee et al., 2020). Upon modelling direct effects of all associations between indicator variables within the best-fitting model, the model became highly unstable, especially if fitted to a relatively small sample. As such we decided to buffer the risk of making a type I error by conducting validation analyses post-extraction.

# Choice of Validation Strategies

With LPA results allowing for various interpretations, inclusion of a validation strategy is crucial. In this study, we focused on subtype separation, rather than cross-method replication, due to difficulties in collecting large volumes of questionnaire data from hospitalized patients from specific diagnostic groups. As such, our choice of validation strategies was guided by the aim to assess the extent to which the profiles we extracted were different enough from each other to appear across different subsets of our data.

Firstly, we report the mean posterior probabilities of class membership across extracted profiles. This approach allows us to quantify classification quality beyond entropy.

Our second validation strategy was the alternative instrument technique in which we related class membership to instruments designed to measure constructs similar to those assessed by our indicator variables post-extraction of profiles. Comparison of profiles across measures not included in construction of subtypes allows for indirect evidence of not the measures themselves but the measured constructs being important to profile extraction. Although equivalent instruments could have also been used to construct subtypes, in our case, missing data caused problems for reliable class identification.

Thirdly, we believe comparative investigation of models with a different number of extracted classes to also support correct identification of profiles.

# BCH versus One-Step Covariate Analyses

In previous research, several methods for including covariates and distal outcomes in analyses have been proposed. In this study, we utilize both the one-step method and the BCH approach (Bolck et al., 2004). In the one-step technique, most probable class membership of each individual is assessed and a factor variable with class membership related to outcome analyses. This approach does not retain the classification uncertainty and is thus less precise, however, with entropy values surpassing .80, it is considered acceptable (Clark and Muthén, 2009). Via creation of one factor variable, we had more opportunities to tailor our analyses to fit the characteristics of or non-normally distributed data. Alongside this technique, we used the BCH approach which makes use of individual class probabilities to allow for profile assignment uncertainty to remain in the solution (Masyn, 2017). However, this approach has been shown to be unreliable with non-normal outcome measures and its computational complexity can render analyses on small samples unfeasible. For comparative purposes, in this paper, we report the BCH approach in Supplementary Materials (S4).

# References

Bolck, A., Croon, M., and Hagenaars, J. (2004). Estimating latent structure models with categorical variables: one-step versus three-step estimators. *Political Anal*. 12, 3–27. doi:10.1093/pan/mph001.

Clark S. L., and Muthén, B. (2009). Relating Latent Class Analysis Results to Variables Not Included in the Analysis. http://www.statmodel.com/download/relatinglca.pdf [Accessed September 5, 2022].

Ferguson, S. L., Moore, E. W. G., and Hull, D. M. (2020). Finding latent groups in observed data: A primer on latent profile analysis in Mplus for applied researchers. *Int. J. Behav. Dev*. 44, 458–468. doi:10.1177/0165025419881721

Lee, J., Jung, K., and Park, J. (2020). Detecting Conditional Dependence Using Flexible Bayesian Latent Class Analysis. *Front. Psychol*. 11. doi:10.3389/fpsyg.2020.01987

Masyn, K. E. (2013). “Latent class analysis and finite mixture modeling,” in: *The Oxford handbook of quantitative methods*, ed. T. Little (Oxford: Oxford University Press), 551–611.

Masyn, K. E. (2017). Measurement invariance and differential item functioning in latent class analysis with stepwise multiple indicator multiple cause modeling. *Struct. Equ. Model*. 24, 180–197. doi:10.1080/10705511.2016.1254049

Morgan, G. B. (2015). Mixed mode latent class analysis: An examination of fit index performance for classification. *Struct. Equ. Model.* 22, 76–86. doi:10.1080/10705511.2014.935751

Muthén, L. K., and Muthén, B. O. (1998–2012). *Mplus User’s Guide, seventh ed*. Los Angeles: Muthén & Muthén.
