# Supplementary material for: Bottom-up transdiagnostic personality subtypes are associated with state psychopathology: A latent profile analysis
Source: Front Psychol. 2023 Feb 21;14:1043394. doi: 10.3389/fpsyg.2023.1043394 (PMC9990091; doi:10.3389/fpsyg.2023.1043394)
Supplement: SUPPLEMENTARY TABLE 2 — S2_Descriptive_Statistics [file Table_2.docx]

Supplementary Material S2

Descriptive Statistics

**Supplementary Table 1. Means, standard deviations and comparisons of means tests for indicator and validation variables across samples**

| Measured variable |  | Subsample | | | ANOVA & Kruskal-Wallis H | | | | Total *N* = 427 |  |
| --- | --- | --- | --- | --- | --- | --- | --- | --- | --- | --- |
|  |  | Control (*n* = 114) | ED (*n* = 249) | MOOD-SUD (*n* = 64) |  |  |  |  |  |  |
|  | *n* | *M*(*SD*) | *M*(*SD*) | *M*(*SD*) | *df* | *F* | *H*(2) | *M*(*SD*) | | |
| Somatic trait anxiety | 407 | 53.3 (9.6)^b, c^ | 60.7 (11.4)^a^ | 63.8 (11.03)^a^ | 2, 404 | 23.55*** | — | 59.09(11.46) | | |
| Psychic trait anxiety | 407 | 49.1 (9.7)^b, c^ | 60.5 (10.9)^a^ | 60.1 (10.82)^a^ | 2, 404 | 46.27*** | — | 57.33(11.69) | | |
| Stress susceptibility | 407 | 49.5 (8.7)^b, c^ | 59.4 (12.1)^a^ | 61.1 (10.40)^a^ | 2, 131 | 46.97^d^*** | — | 56.92(11.94) | | |
| Detachment | 407 | 45.0 (8.8)^b, c^ | 51.5 (9.8)^a^ | 50.2 (10.31)^a^ | 2, 404 | 17.49*** | — | 49.56(9.97) | | |
| Embitterment | 407 | 49.6 (9.5) ^b, c^ | 60.3 (11.8)^a^ | 61.6 (10.99)^a^ | 2, 404 | 40.17*** | — | 57.61(12.13) | | |
| Trait irritability | 407 | 51.5 (8.6)^b, c^ | 55.8 (9.5)^a^ | 57.1 (9.64)^a^ | 2, 404 | 9.66*** | — | 54.82(9.75) | | |
| Mistrust | 407 | 51.9 (10.0)^b, c^ | 59.0 (11.2)^a^ | 59.6 (14.52)^a^ | 2, 131 | 18.85^d^*** | — | 57.16(11.81) | | |
| Positive perfectionism | 381 | 32.7 (9.2)^b^ | 38.3 (9.9)^a, c^ | 34.2 (10.20)^b^ | 2, 378 | 13.98*** | — | 36.24(9.82) | | |
| Negative perfectionism | 381 | 10.7 (8.6)^b, c^ | 21.8 (13.4)^a^ | 25.9 (11.7)^a^ | 2, 145 | 56.45^d^*** | — | 19.42(13.20) | | |
| Functional impulsivity | 395 | 26.3 (7.8)^b, c^ | 20.7 (8.2)^a^ | 19.7 (8.4)^a^ | 2, 392 | 18.73*** | — | 21.85(8.49) | | |
| Dysfunctional impulsivity | 396 | 13.5 (6.3)^b, c^ | 16.6 (7.7)^a ,c^ | 19.5 (8.0)^a, b^ | 2, 143 | 14.14^d^*** | — | 16.32(7.70) | | |
| BIS-11 impulsivity | 275 | 53.1 (9.7)^b, c^ | 61.0 (11.5)^a^ | 63.3 (11.9)^a^ | 2, 272 | 13.52*** | — | 59.86(11.74) | | |
| Trait anxiety | 279 | 31.7 (9.2)^b, c^ | 55.8 (11.9)^a^ | 53.3 (14.7)^a^ | 2, 111 | 74.34^d^*** | 73.78 | 51.41(14.34) | | |
|  |  |  |  |  |  |  |  | *(cont.)* | | |
| **Supplementary Table 1 cont.** | | |  |  |  |  |  |  |  |  |
|  |  | Subsample | | | ANOVA & Kruskal-Wallis H | | | | Total *N* = 427 |  |
| Measured variable |  | Control (*n* = 114) | ED (*n* = 249) | MOOD-SUD (*n* = 64) |  |  |  |  |  |  |
|  | *n* | *M*(*SD*) | *M*(*SD*) | *M*(*SD*) | *df* | *F* | *H*(2) | *M*(*SD*) | | |
| Neuroticism^e^ | 133 | 70.1 (21.9) | 109.8 (29.5) | N/A | 130 | 8.91*** | — | 92.2(33.0) | | |
| Extraversion^e^ | 133 | 124.0 (17.0) | 99.8 (24.5) | N/A | 129 | –6.71*** | — | 110.5(24.6) | | |
| Agreeableness^e^ | 133 | 133.3 (15.6) | 129.6 (23.0) | N/A | 128 | –1.10*** | — | 131.3(20.1) | | |
| Conscientiousness^e^ | 133 | 129.6 (21.6) | 122.8 (26.6) | N/A | 131 | –1.61*** | — | 125.8(24.67) | | |
| Depression | 285 | 6.4 (4.6)^b, c^ | 19.7 (10.3)^a^ | 20.5 (11.0)^a^ | 2, 138 | 105.2^d^*** | — | 17.3(11.0) | | |
| Emotion regulation difficulties | 165 | 21.3 (20.2)^b, c^ | 90.8 (24.0)^a^ | 92.0 (26.0)^a^ | 2, 162 | 86.17*** | — | 81.0(34.5) | | |
| State anxiety | 282 | 31.4 (9.2)^b, c^ | 50.4 (14.3)^a^ | 47.2 (15.9)^a^ | 2, 125 | 67.01^d^*** | 64.43 | 46.0(15.6) | | |
| Dietary restraint | 419 | 11.8 (6.9)^b^ | 22.8 (10.1)^a, c^ | 11.1 (8.0)^b^ | 2, 167 | 87.42^d^*** | 115.76 | 18.1(10.6) | | |
| Purging | 419 | 0.3 (1.2)^b, c^ | 7.5 (7.4)^a, c^ | 1.7 (3.0)^a, b^ | 2, 148 | 125.36^d^*** | 143.66 | 4.6(6.2) | | |
| Preoccupation with weight | 420 | 9.1 (7.46)^b, c^ | 24.1 (11.1)^a, c^ | 16.5 (11.9)^a, b^ | 2, 153 | 114.00^d^*** | 124.81 | 18.9(12.2) | | |

*Note.* All *post-hoc* tests significant at *p* < .05. ED = eating disorder sample, MOOD-SUD = mood, anxiety and substance use disorder sample. BIS11 = Barratt Impulsiveness Scale.

^a^ Statistically significantly different from the control sample. ^b^ Different from the ED sample. ^c^ Different from the MOOD-SUD sample. ^d^ Welsch correction and Games-Howell test applied. ^e^ t-tests conducted and the t-statistic reported.

** p* < .001
