# Supplementary material for: Bottom-up transdiagnostic personality subtypes are associated with state psychopathology: A latent profile analysis
Source: Front Psychol. 2023 Feb 21;14:1043394. doi: 10.3389/fpsyg.2023.1043394 (PMC9990091; doi:10.3389/fpsyg.2023.1043394)
Supplement: SUPPLEMENTARY TABLE 3 — S3_Model_Fit_Statistics [file Table_3.docx]

Supplementary Material S3

Latent Profile Analysis Model Fit Statistics

**Supplementary Table 2. Fit indices for 1–8 class LPA models in the total clinical sample**

| No | LL | AIC | BIC | SABIC | Entropy | SMALL % | LMR (*p*) | BLRT (*p*) |
| --- | --- | --- | --- | --- | --- | --- | --- | --- |
| 1 | –12,176.77 | 24,397.54 | 24,479.46 | 24,409.69 | — | — | — | — |
| 2 | –11,843.89 | 23,755.77 | 23,882.38 | 23,774.54 | .80 | 42.8% | .10 | <*.*001 |
| 3 | –11,680.11 | 23,452.22 | 23,623.50 | 23,477.61 | .88 | 12.8% | <*.*001 | <*.*001 |
| 4 | –11,633.31 | 23,382.63 | 23,598.60 | 23,414.65 | .81 | 10.8% | .34 | <*.*001 |
| 5 | –11,602.77 | 23,345.54 | 23,606.19 | 23,384.18 | .77 | 10.1% | .64 | <*.*001 |
| 6 | –11,572.38 | 23,308.77 | 23,614.10 | 23,354.04 | .81 | 2.9% | .31 | <*.*001 |
| 7 | –11,545.70 | 23,279.39 | 23,629.41 | 23,331.28 | .82 | 3.6% | .46 | <*.*001 |
| 8 | –11,519.82 | 23,251.64 | 23,646.34 | 23,310.15 | .82 | 3.6% | .51 | <*.*001 |

*Note.* No = Number of classes in tested model; LL = log likelihood; AIC = Akaike’s Information Criterion; BIC = Bayesian Information Criterion, SABIC = sample-adjusted Bayesian information criterion; ENT = entropy, SMALL % = smallest class %; LMR *p* = adjusted Lo-Mendell-Rubin likelihood ratio test; BLRT = bootstrap likelihood ratio test.

**Supplementary Table 3. Fit indices for 1–8 class LPA models in the control sample**

| No | LL | AIC | BIC | SABIC | ENT | SMALL % | LMR (*p*) | BLRT (*p*) |
| --- | --- | --- | --- | --- | --- | --- | --- | --- |
| 1 | –4,178.07 | 8,400.14 | 8,460.34 | 8,390.80 | — | — | — | — |
| 2 | –4,067.34 | 8,202.68 | 8,295.71 | 8,188.25 | .91 | 32.5% | <.01 | <.01 |
| 3 | –4,029.97 | 8,151.94 | 8,277.81 | 8,132.42 | .88 | 13.8% | .07 | <.01 |
| 4 | –3,994.08 | 8,104.15 | 8,262.85 | 8,079.53 | .90 | 5.3% | .16 | <.01 |
| 5 | –3,978.24 | 8,096.48 | 8,288.01 | 8,066.77 | .92 | 5.3% | .78 | .50 |
| 6 | –3,965.77 | 8,095.54 | 8,319.91 | 8,060.73 | .92 | 1.8% | .36 | 1.00 |
| 7 | –3,949.05 | 8,086.09 | 8,343.30 | 8,046.19 | .90 | 1.8% | .77 | .33 |
| 8 | –3,933.97 | 8,079.94 | 8,369.98 | 8,034.95 | .91 | 1.8% | .76 | . 60 |

*Note.* For analyses with different numbers of starts, the relative order of all fit indices remained the same, with the exception of a larger number of starting values favoring the 8-class solution based on AIC and SABIC. No = Number of classes in tested model; LL = log likelihood; AIC = Akaike’s Information Criterion; BIC = Bayesian Information Criterion; SABIC = sample-adjusted Bayesian information criterion; ENT = entropy, SMALL % = smallest class %; LMR = adjusted Lo-Mendell-Rubin likelihood ratio test; BLRT = bootstrap likelihood ratio test.

**Supplementary Table 4. Fit indices for 1–8 class LPA models in the ED sample**

| No | LL | AIC | BIC | SABIC | ENT | SMALL % | LMR (*p*) | BLRT (*p*) |
| --- | --- | --- | --- | --- | --- | --- | --- | --- |
| 1 | –9,852.55 | 19,749.11 | 19,826.13 | 19,756.40 | — | — | — | — |
| 2 | –9,569.06 | 19,206.12 | 19,325.17 | 19,217.39 | .83 | 36.3% | .08 | <.01 |
| 3 | –9,427.21 | 18,946.42 | 19,107.47 | 18,961.66 | .88 | 13.9% | .01 | <.01 |
| 4 | –9,382.72 | 18,881.48 | 19,084.55 | 18,900.69 | .87 | 4.9% | .39 | <.01 |
| 5 | –9,351.50 | 18,843.01 | 19,088.10 | 18,866.20 | .84 | 4.5% | .67 | <.01 |
| 6 | –9,325.07 | 18,814.14 | 19,101.24 | 18,841.31 | .83 | 4.1% | .51 | <.01 |
| 7 | –9,298.68 | 18,785.25 | 19,114.37 | 18,816.40 | .84 | 3.3% | .52 | <.01 |
| 8 | –9,277.36 | 18,766.73 | 19,137.86 | 18,801.85 | .85 | 2.9% | .31 | <.01 |
| *Note.* No = Number of classes in tested model; LL = log likelihood; AIC = Akaike’s Information Criterion; BIC = Bayesian Information Criterion, SABIC = sample-adjusted Bayesian information criterion; ENT = entropy, SMALL % = smallest class %; LMR *p* = adjusted Lo-Mendell-Rubin likelihood ratio test; BLRT = bootstrap likelihood ratio test. | | | | | | | | |

**Supplementary Table 5. Fit indices for 1–8 class LPA models in the MOOD-SUD sample**

| No | LL | AIC | BIC | SABIC | ENT | SMALL % | LMR (*p*) | BLRT (*p*) |
| --- | --- | --- | --- | --- | --- | --- | --- | --- |
| 1 | –2,305.91 | 4,655.83 | 4,792.27 | 4,633.06 | — | — | — | — |
| 2 | –2,233.29 | 4,534.58 | 4,606.35 | 4,499.40 | .89 | 27.9% | .03 | <.01 |
| 3 | –2,207.68 | 4,507.36 | 4,604.46 | 4,459.75 | .86 | 23.0% | .66 | <.01 |
| 4 | –2,190.03 | 4,496.06 | 4,618.49 | 4,436.04 | .87 | 11.5% | .45 | .04 |
| 5 | –2,173.36 | 4,486.72 | 4,634.48 | 4,414.27 | .91 | 4.9% | .24 | .16 |
| 6 | –2,155.59 | 4,475.18 | 4,648.27 | 4,390.32 | .91 | 6.6% | .76 | .20 |
| 7 | –2,141.46 | 4,470.92 | 4,669.35 | 4,373.64 | .92 | 4.9% | .61 | .50 |
| 8 | –2,123.31 | 4,458.62 | 4,682.37 | 4,348.92 | .92 | 4.9% | .84 | .18 |

*Note.* No = Number of classes in tested model; LL = log likelihood; AIC = Akaike’s Information Criterion; BIC = Bayesian Information Criterion, SABIC = sample-adjusted Bayesian information criterion; ENT = entropy, SMALL % = smallest class %; LMR *p* = adjusted Lo-Mendell-Rubin likelihood ratio test; BLRT = bootstrap likelihood ratio test.
