# Supplementary material for: Bottom-up transdiagnostic personality subtypes are associated with state psychopathology: A latent profile analysis
Source: Front Psychol. 2023 Feb 21;14:1043394. doi: 10.3389/fpsyg.2023.1043394 (PMC9990091; doi:10.3389/fpsyg.2023.1043394)
Supplement: SUPPLEMENTARY TABLE 4 — S4_Five-Profile_Model_Statistics [file Table_4.docx]

Supplementary Material S4

5-Profile Model Characteristics

**Supplementary Table 6. Comparison of means of indicator variables across profiles in the best-fitting total sample 5-profile model**

|  | Profile | | | | | ANOVA | | | | |
| --- | --- | --- | --- | --- | --- | --- | --- | --- | --- | --- |
| Indicator variable | High-Functioning (*n* = 56) | Well-Adapted (*n* = 112) | Impulsive and Interpersonally Dysregulated (*n* = 114) | Anxious and Perfectionistic (*n* = 66) | Emotionally and Behaviorally Dysregulated (*n* = 72) | *df2* | | *F* | ω^2^ | 95%  for ω^2^ |
|  | *M*(*SD*) | *M*(*SD*) | *M*(*SD*) | *M*(*SD*) | *M*(*SD*) |  | | | | |
| Somatic anxiety | 44.2(7.0)^b, c, d, e^ | 52.3(7.1)^a, c, d, e^ | 63.2(7.6)^a, b, e^ | 63.3(8.8)^a, b, e^ | 70.7(8.4)^a, b, c, d^ | 175 | 125.65*^f^ | | .73 | [.68, .77] |
| Psychic anxiety | 39.2(5.9)^b, c, d, e^ | 52.6(6.7)^a, c, d, e^ | 55.7(6.2)^a, b, d, e^ | 67.7(4.2)^a, b, c, e^ | 71.5(6.2)^a, b, c, d^ | 180 | 326.30*^f^ | | .88 | [.85, .89] |
| Stress susceptibility | 40.7(6.9)^b, c, d, e^ | 50.4(6.7) ^a, c, d, e^ | 57.0(7.2)^a, b, d, e^ | 65.2(7.4)^a, b, c, e^ | 71.4(7.6)^a, b, c, d^ | 402 | 185.06* | | .64 | [.60, .68] |
| Detachment | 40.3(8.0)^b, c, d, e^ | 45.9(8.1)^a, c, e^ | 53.3(8.3)^a, b, d, e^ | 49.0(7.9)^a, c, e^ | 56.9(10.3)^a, b, c, d^ | 402 | 39.40* | | .21 | [.15, .26] |
| Embitterment | 42.3(6.1)^b, c, c, e^ | 49.8(5.9)^a, c, d, e^ | 60.9(6.8)^a, b, e^ | 59.9(6.8)^a, b, e^ | 75.1(8.1)^a, b, c, d^ | 175 | 208.84*^f^ | | .82 | [.79, .85] |
| Trait Irritability | 43.9(7.4)^b, c, d, e^ | 50.3(7.5)^a, c, d, e^ | 57.2(7.9)^a, b, e^ | 57.2(7.7)^a, b, e^ | 64.2(8.4)^a, b, c, d^ | 402 | 68.57* | | .37 | [.32, .43] |
| Mistrust | 42.5(6.7)^b, c, d, e^ | 51.3(8.3)^a, c, d, e^ | 60.9(7.9)^a, b, d, e^ | 57.5(8.0)^a, b, c, e^ | 71.1(9.4)^a, b, c, d^ | 402 | 112.48*^g^ | | .45 | [.40, .50] |
| Positive perfectionism | 30.4(10.2)^b, d, e^ | 36.8(9.7)^a, c, e^ | 32.7(8.4)^b, d, e^ | 40.2(8.2)^a, c^ | 42.0(8.5)^a, b, c^ | 376 | 19.02* | | .10 | [.06, .15] |
| Negative perfectionism | 6.2(7.7)^b, c, d, e^ | 13.0(10.1)^a, c, d, e^ | 19.8(10.9)^a, b, e^ | 24.2(9.7)^a, b, e^ | 34.8(9.2)^a, b, c, d^ | 172 | 95.30*^f, h^ | | .62 | [.62, .73] |
| Functional impulsivity | 30.2(7.4)^b, c, d, e^ | 24.1(7.1)^a, d, e^ | 22.3(7.4)^a, d, e^ | 15.6(6.2)^a, b, c^ | 17.5(8.0)^a, b, c^ | 390 | 37.73* | | .23 | [.18, .29] |
|  |  |  |  |  |  |  |  | | *(cont.)* | |

**Supplementary Table 6 cont.**

|  | Profile | | | | | ANOVA | | | | |
| --- | --- | --- | --- | --- | --- | --- | --- | --- | --- | --- |
| Indicator variable | High-Functioning (*n* = 56) | Well-Adapted (*n* = 112) | Impulsive and Interpersonally Dysregulated (*n* = 114) | Anxious and Perfectionistic (*n* = 66) | Emotionally and Behaviorally Dysregulated (*n* = 72) | *df2* | | *F* | ω^2^ | 95%  for ω^2^ |
|  | *M*(*SD*) | *M*(*SD*) | *M*(*SD*) | *M*(*SD*) | *M*(*SD*) |  | | | | |
| Dysfunctional impulsivity | 11.6(6.5)^c, e^ | 13.4(5.8)^c, e^ | 18.9(5.7)^a, b, d, e^ | 12.5(5.9)^c, e^ | 23.5(8.6)^a, b, c, d^ | 168 | 36.34*^f, i^ | | .45 | [.35, .52] |

*Note.* Tukey test applied, *post-hoc* tests significant at *p* < .05. For all ANOVA tests, *df1* = 4.

^a^ Statistically significantly different from high-functioning profile. ^b^ Different from well-adapted profile. ^c^ Different from impulsive and interpersonally dysregulated profile. ^d^ Different from anxious and perfectionistic profile. ^e^ Different from emotionally and behaviorally dysregulated profile. ^f^ Welch ANOVA, Games-Howell test applied, effect size is an estimation. ^g^ Non-normal distribution; *H*(4) = 214.81 ^h^ *H*(4) = 172.09 ^i^ *H*(4) = 115.06.

**p* < .001

**Supplementary Table 7. Comparison of means of alternative instruments and distal outcomes across profiles in the best-fitting total sample 5-profile model using the BCH approach**

| Variable | Class | | | | | BCH *χ^2^* |
| --- | --- | --- | --- | --- | --- | --- |
|  | High-Functioning (*n* =56) | Well-Adapted (*n* = 112) | Impulsive and Interpersonally Dysregulated (*n* = 114) | Anxious and Perfectionistic (*n* = 66) | Emotionally and Behaviorally Dysregulated (*n* = 72) |  |
|  | *M*(*SE*) | *M*(*SE*) | *M*(*SE*) | *M*(*SE*) | *M*(*SE*) |  |
| Alternative instruments |  |  |  |  |  |  |
| Impulsivity | 53.0 (1.7)^c, e^ | 53.7 (1.6)^c, e^ | 64.8 (1.4)^a, b, d^ | 56.5 (1.8)^c, e^ | 68.3 (2.0)^a, b, d^ | 66.93* |
| Trait anxiety | 33.4 (2.2)^b, c, d, e^ | 42.8 (1.7)^a, c, d, e^ | 53.5 (1.6)^a, b, d, e^ | 59.3 (1.7)^a, b, c, e^ | 64.4 (1.7)^a, b, c, d^ | 198.67* |
| Conscientiousness | 143.2 (4.7)^c, e^ | 136.1 (3.2)^c, e^ | 111.5 (5.0)^a, b, d^ | 132.1 (6.1)^c, e^ | 101.9 (6.1)^a, b, d^ | 47.30* |
| Neuroticism | 51.7 (5.9)^b, c, d, e^ | 77.6 (3.1)^a, c, d, e^ | 95.6 (4.0)^a, b, d, e^ | 118.0 (7.1)^a, b, c, e^ | 141.8 (3.7)^a, b, c, d^ | 275.16* |
| Agreeableness | 143.5 (2.5)^b, c, e^ | 135.2 (2.1)^a, c, d, e^ | 120.0 (4.9)^a, b, d^ | 150.2 (4.4)^b, c, e^ | 113.0 (6.2)^a, b, d^ | 40.97* |
| Extraversion | 122.5 (4.8)^c, d, e^ | 119.7 (3.4)^d, e^ | 108.8 (4.8)^a, e^ | 96.1 (8.8)^a, b^ | 91.0 (6.2)^a, b, c^ | 28.04* |
| Distal outcomes |  |  |  |  |  |  |
| Depression | 4.9 (1.1)^b, c, d, e^ | 12.0 (1.4)^a, c, d, e^ | 18.2 (1.3)^a, b, e^ | 20.5 (1.5)^a, b, e^ | 28.1 (1.5)^a, b, c, d^ | 213.47* |
| State anxiety | 29.4 (2.1)^b, c, d, e^ | 39.5 (2.2)^a, c, d, e^ | 47.3 (1.8)^a, b, e^ | 51.1 (2.3)^a, b, e^ | 59.1 (2.0)^a, b, c, d^ | 130.48* |
| Emotion regulation | 27.4 (5.8)^b, c, d, e^ | 57.3 (4.7)^a, c, d, e^ | 86.4 (6.4)^a, b, e^ | 94.9 (3.8)^a, b, e^ | 113.6 (3.6)^a, b, c, d^ | 223.70* |
| Restrained eating | 10.1 (1.7)^b, c, d, e^ | 16.2 (1.1)^a, d, e^ | 16.7 (1.2)^a, d, e^ | 23.5 (1.6)^a, b, c^ | 24.2 (1.2)^a, b, c^ | 94.47* |
| Binge eating | 7.9 (1.1)^b, c, d, e^ | 13.3 (1.0)^a, c, d, e^ | 19.4 (1.1)^a, b^ | 17.5 (1.6)^a, b^ | 20.5 (1.6)^a, b^ | 82.91* |
| Purging | 1.3 (0.6)^c, d, e^ | 2.7 (0.6)^c, d, e^ | 5.8 (0.8)^a, b, e^ | 4.9 (0.9)^a, b, e^ | 8.2 (0.9)^a, b, c, d^ | 62.86* |
|  |  |  |  |  | *(cont.)* | |
| **Supplementary Table 7 cont.** | | | | | |  |
| Variable | Class | | | | | BCH *χ^2^* |
|  | High-Functioning (*n* =56) | Well-Adapted (*n* = 112) | Impulsive and Interpersonally Dysregulated (*n* = 114) | Anxious and Perfectionistic (*n* = 66) | Emotionally and Behaviorally Dysregulated (*n* = 72) |  |
|  | *M*(*SE*) | *M*(*SE*) | *M*(*SE*) | *M*(*SE*) | *M*(*SE*) |  |
| Preoccupation with weight | 8.4 (1.4)^b, c, d, e^ | 13.4 (1.2)^a, c, d, e^ | 19.1 (1.3)^a, b, d, e^ | 25.9 (1.7)^a, b, c^ | 29.0 (1.2)^a, b, c^ | 188.45* |

*Note. Post-hoc* tests significant at *p* < .05.

^a^ Statistically significantly different than high-functioning profile. ^b^ Different than well-adapted profile. ^c^ Different than impulsive and interpersonally dysregulated profile. ^d^ Different than anxious and perfectionistic profile. ^e^ Different than emotionally and behaviorally dysregulated profile.

**p* < .001.
