# Supplementary material for: Bottom-up transdiagnostic personality subtypes are associated with state psychopathology: A latent profile analysis
Source: Front Psychol. 2023 Feb 21;14:1043394. doi: 10.3389/fpsyg.2023.1043394 (PMC9990091; doi:10.3389/fpsyg.2023.1043394)
Supplement: SUPPLEMENTARY TABLE 5 — S5_Subsample_Profiles_Graphically [file Table_5.docx]

Supplementary Material S5

Subsample Profile Solutions Presented Graphically

**Supplementary Figure 1. Z-scores with 95% confidence intervals in the 5-profile total clinical sample with initial class labels**


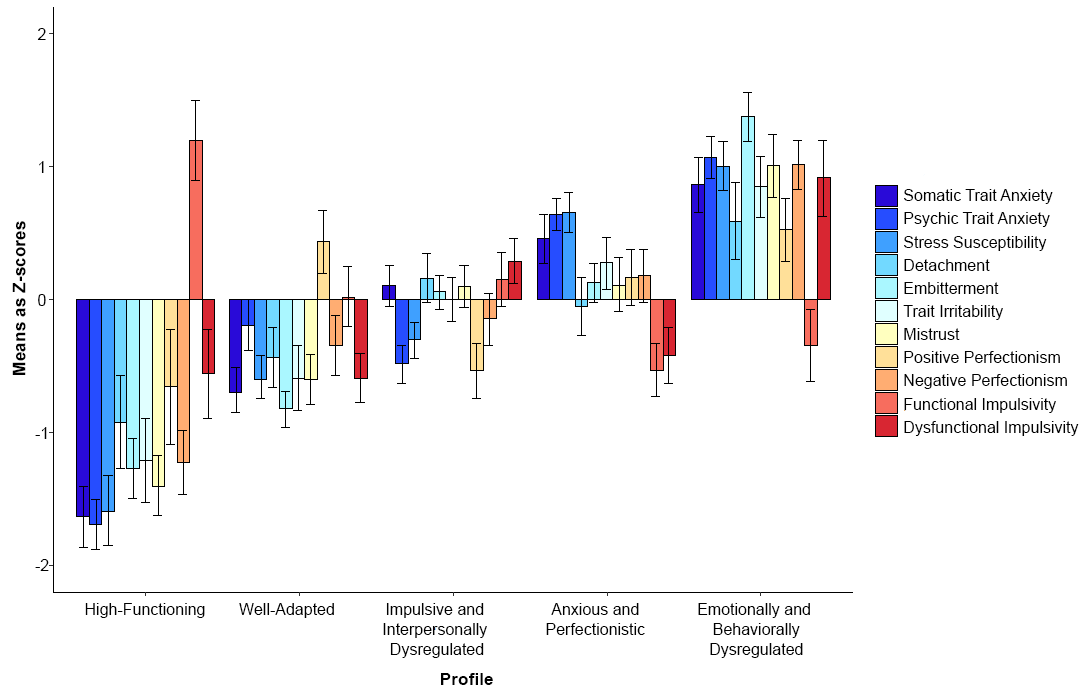


*Note.* 5-class model entropy was .77. Number of people assigned to high-functioning profile is *n* = 31, to well-adapted profile *n* = 60, impulsive and interpersonally dysregulated *n* = 88, anxious and perfectionistic *n* = 66, emotionally and behaviorally dysregulated *n* = 61.

**Supplementary Figure 2. Z-scores with 95% confidence intervals in the 5-profile control sample with initial class labels**

*
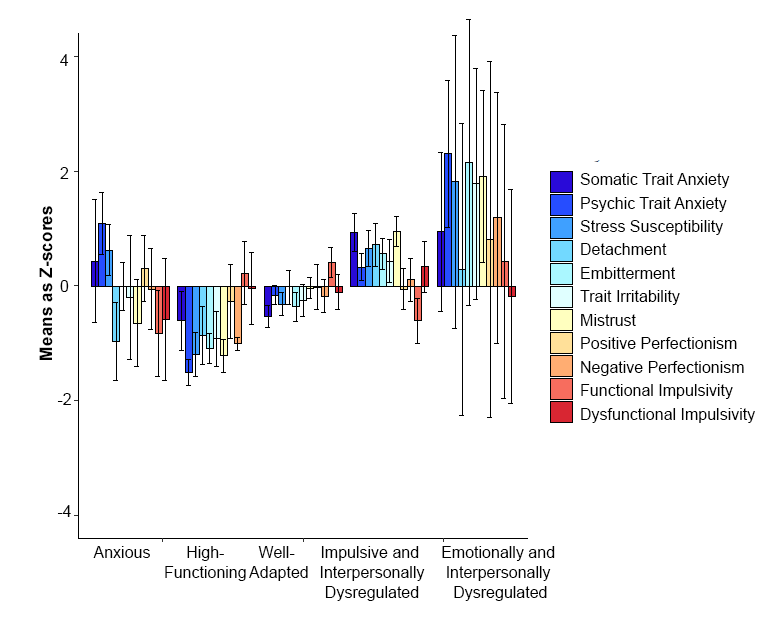
*

*Note.* 5-class model entropy was .92. Number of people assigned to anxious profile is *n* = 13, to high-functioning profile *n* = 19, well-adapted *n* = 49, impulsive and interpersonally dysregulated *n* = 27, emotionally and interpersonally dysregulated *n* = 6.

**Supplementary Figure 3. Z-scores with 95% confidence intervals in the 5-profile ED sample with initial class labels**


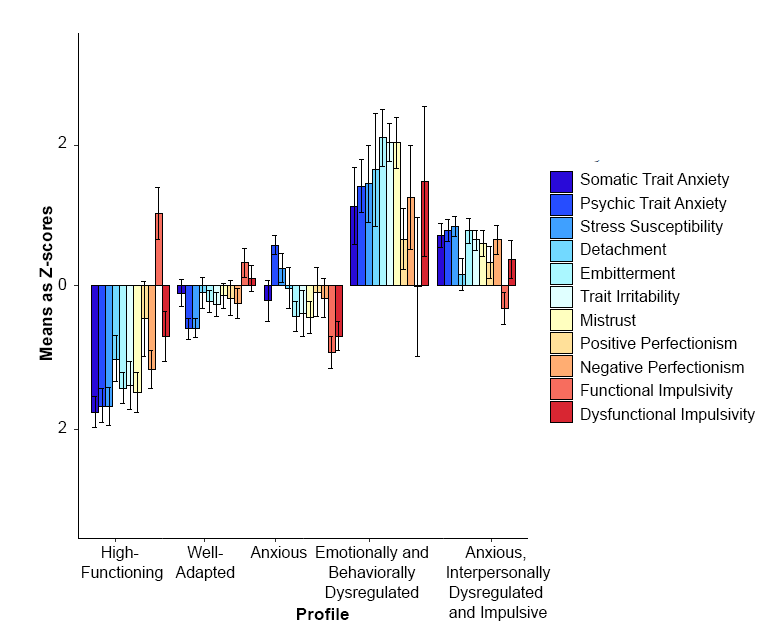


*Note.* 5-class model entropy was .84. Number of people assigned to high-functioning profile is *n* = 29, to well-adapted profile *n* = 86, anxious *n* = 42, emotionally and behaviorally dysregulated *n* = 11, anxious, interpersonally dysregulated and impulsive *n* = 77.

**Supplementary Figure 4. Z-scores with 95% confidence intervals in the 5-profile MOOD-SUD sample with initial class labels**


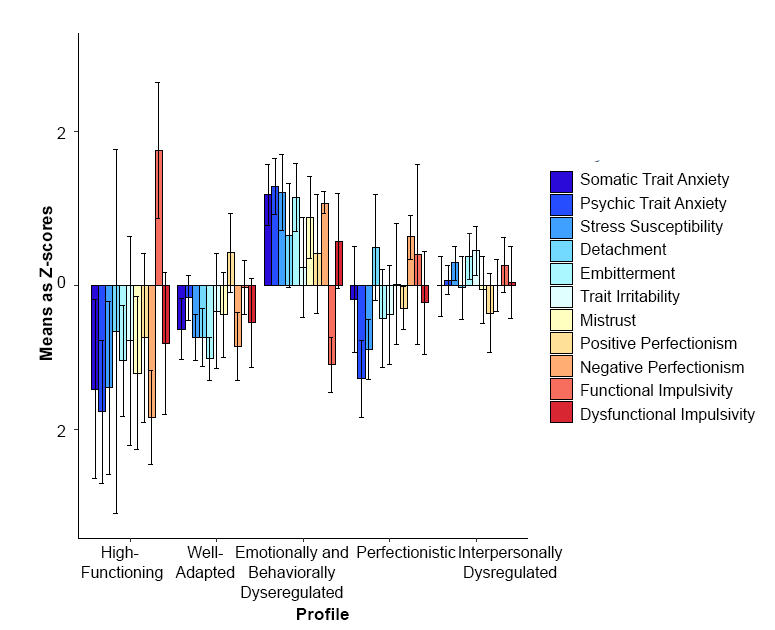


*Note.* 5-class model entropy was .91. Number of people assigned to high-functioning profile is *n* = 4, to well-adapted profile *n* = 16, emotionally and behaviorally dysregulated *n* = 14, perfectionistic *n* = 7, interpersonally dysregulated *n* = 20.
